# The Application Value of Nursing Interventions Based on the Chronic Illness Trajectory Framework in Patients With Amyotrophic Lateral Sclerosis

**DOI:** 10.1155/nri/1280057

**Published:** 2026-01-30

**Authors:** Jiao Zhen, Qia Liu, Xiaolu Xue, Fengjiao Hao, Shufang Zhang, Na Liu, Zixuan Li, Jing Chen, Junxiang Cheng

**Affiliations:** ^1^ Department of Neurology, The First Hospital of Shanxi Medical University, Taiyuan, 030001, China, sxmu.edu.cn; ^2^ Nursing College of Shanxi Medical University, Taiyuan, 030001, China; ^3^ Department of Neurology, Shanxi Cardiovascular Disease Hospital, Taiyuan, 030001, China; ^4^ Department of Plastic, Aesthetic, and Burn Surgery, The First Hospital of Shanxi Medical University, Taiyuan, 030001, China, sxmu.edu.cn; ^5^ Department of Mental Health, The First Hospital of Shanxi Medical University, Taiyuan, 030001, China, sxmu.edu.cn

**Keywords:** amyotrophic lateral sclerosis, chronic illness trajectory framework, nursing interventions

## Abstract

This prospective study evaluated the impact of nursing interventions based on the Chronic Illness Trajectory Framework (CITF) on anxiety, depression, mental toughness, sleep quality, and ALSFRS‐R scores in amyotrophic lateral sclerosis (ALS) patients to enhance care strategies. Eighty ALS patients were enrolled from the Department of Neurology at the First Hospital of Shanxi Medical University between February 2023 and March 2024. Participants were randomly assigned to an intervention group (CITF‐based nursing interventions) or a control group (standard care). Over an 8‐week period, the intervention group demonstrated significantly lower anxiety and depression scores, higher mental toughness, and improved sleep quality compared to the control group (*p* < 0.05). Additionally, the intervention group achieved higher ALSFRS‐R scores (31.63 ± 3.54 vs. 29.58 ± 3.38) (*p* < 0.05). These findings indicate that CITF‐based nursing interventions effectively reduce negative emotional states, enhance mental resilience, improve sleep quality, and boost overall quality of life in ALS patients. Based on this study, nurses can integrate CITF‐based interventions into standard ALS care to enhance patients’ emotional well‐being and functional outcomes.

**Trial Registration:** Chinese Clinical Trial Registry: ChiCTR2500108691

**Keypoints**
• Effective management of emotional well‐being
◦ CITF‐based nursing interventions significantly reduce anxiety and depression levels in ALS patients, enhancing their emotional stability during disease progression.
• Enhanced psychological resilience and sleep quality
◦ Patients receiving CITF‐based care demonstrate increased mental toughness and improved sleep quality, contributing to a better overall quality of life.
• Improved functional outcomes
◦ The intervention group achieved higher ALSFRS‐R scores, indicating better functional status and suggesting that CITF‐based nursing strategies can positively influence disease management in ALS.

## 1. Introduction

Amyotrophic lateral sclerosis (ALS) is a neurodegenerative disorder marked by the gradual deterioration and loss of both upper and lower motor neurons. Clinical manifestations include progressive muscle weakness and atrophy, with most patients dying within approximately 3–5 years because of respiratory failure [1]. The annual incidence of ALS has been reported to range from 0.6 to 3.8 cases per 100,000 individuals [2]. In Europe, ALS incidence was higher, with rates between 2.1 and 3.8 cases per 100,000 individuals [3, 4]. It was estimated that nearly half a million people worldwide suffer from ALS, with the majority of patients being diagnosed between the ages of 40 and 70. In addition to muscle weakness and atrophy [5], ALS patients often experience severe negative emotions, which further reduce their quality of life and treatment compliance. Nurses should provide comprehensive care and essential support to patients. High‐quality nursing interventions and compassionate care help alleviate negative emotions, playing a crucial role throughout the treatment for ALS [6].

The concept of illness trajectory initially described the events influenced by the response to the condition, interactions with others, and various interventions. This concept has been widely described in the contexts of chronic illness and the dying process. Subsequently, Corbin and Strauss proposed the Chronic Illness Trajectory Framework (CITF), which divides the disease process into the following stages: pretrajectory, trajectory onset, crisis, acute, stable, unstable, comeback, downward, and dying. Each stage has three dimensions of the theory, including illness‐related work, biographical work, and everyday life work, which can be used for evaluation and intervention [7, 8]. The management model focused on the CITF posits that care for chronic diseases must adapt in response to the progression of the illness. Therefore, the CITF framework customizes interventions for patients with chronic illnesses. It provides education about the disease, fosters a positive self‐image, and encourages self‐care practices in daily life. This theory aimed to help chronic disease patients manage their symptoms, minimize complications, and sustain emotional stability [7, 9]. Research has already developed healthcare interventions based on CITF in multiple diseases such as stroke, breast cancer, and others, yielding favorable application outcomes [10, 11]. However, its application within the context of ALS care remains relatively unexplored and warrants further empirical attention.

Previous research on ALS has mostly focused on the late stage of the disease, including interventions such as multidisciplinary team care, the early use of non‐invasive ventilation, gastrostomy, symptom management, and palliative care [12]. ALS is characterized by a predictable yet rapidly progressive trajectory, with complex needs evolving across the illness continuum. The CITF is uniquely suited to address these needs through its focus on tracking physiological and psychosocial shifts. This framework enables dynamic interventions tailored to specific disease phases, which is essential for ALS care where patient status changes rapidly. Additionally, its emphasis on coordinating care among patients, families, and healthcare teams helps to mitigate the profound dependency and caregiver burden in ALS.

This study systematically developed an ALS nursing intervention based on CITF and investigated its impact on anxiety, depression, mental toughness, sleep quality, and ALSFRS‐R scores among ALS patients. Our research aims to provide a new direction for ALS nursing strategies to enhance psychological stability.

## 2. Materials and Methods

### 2.1. Patients

This prospective study enrolled 80 patients diagnosed with ALS from the Department of Neurology at the First Hospital of Shanxi Medical University between February 2023 and March 2024. An independent investigator used a computer‐generated random number table to randomly assign the patients into two groups, with 40 patients in the intervention group and 40 in the control group. The intervention group received nursing care based on the CITF, while the control group received standard care.

To ensure a double‐blind concealed allocation method, the research team followed these steps: Each patient’s group assignment was stored in a sealed, opaque envelope labeled with the patient’s number and group assignment (intervention or control). These envelopes were prepared and numbered by an independent third party to ensure that group allocation remained unknown prior to the start of the study. When patients met the inclusion criteria and consented to participate, the research team retrieved the group assignment from the envelopes in sequence, and care was delivered accordingly. Throughout the study, neither the researchers nor the patients were informed of the group assignments to avoid any psychological expectations or placebo effects that might bias the outcomes.

#### 2.1.1. Inclusion Criteria

(1) Based on the revised El Escorial criteria [13], patients diagnosed with ALS must meet the clinically definite, probable, or laboratory‐supported criteria. (2) Consciousness and cognitive functions were normal (as defined by a Montreal Cognitive Assessment [MoCA] score of ≥ 26). (3) Patients and their families were briefed on the study’s details and provided written informed consent.

#### 2.1.2. Exclusion Criteria

(1) Patients with severe impairment of liver, kidney, and lung function. (2) Patients with psychiatric disorders or a family history of mental illness. (3) Long‐term living alone or lack of family cooperation in caregiving. This study was approved by the Medical Ethics Committee of the First Hospital of Shanxi Medical University (Approval No. KYLL‐2024‐258).

#### 2.1.3. Sample Size Calculation

The sample size was calculated a priori using G^∗^Power software based on the primary outcome (ALSFRS‐R score) [14]. With an anticipated effect size (Cohen’s d) of 0.65, an alpha of 0.05, and a power of 0.8, a minimum of 38 participants per group was required. A total of 80 participants (40 per group) were enrolled, which provides adequate power for the analysis.

## 3. Methods

All patients received standard ALS pharmacotherapy, including medications such as riluzole, edaravone, and others. The use of these medications was uniform across both the intervention and control groups. Specific drugs, dosages, and frequencies adhered to the Guidelines for the Treatment and Management of ALS.

The control group was provided with standard nursing care, including medication guidance, health education on disease‐related knowledge, dietary control, rehabilitation care, and routine medical follow‐ups. These interventions represent standard practices in ALS management, following the Good Clinical Practice guidelines of the International Conference on Harmonisation and the ethical principles of the Declaration of Helsinki.

In the intervention group, a comprehensive management strategy based on CITF was implemented. This involved establishing a CITF nursing intervention team comprising 7 members, including physicians, rehabilitation therapists, psychologists, and nurses, all of whom are experienced healthcare professionals with over 5 years of clinical expertise in managing ALS. Based on the CITF, nursing interventions for ALS were planned according to the disease trajectory. We adapted the clinical staging system proposed by Roche et al. to categorize the trajectory into three primary phases for nursing purposes [15]: the disease‐onset stage (corresponding to Roche Stage 1: symptom onset), the disease‐diagnosis stage (Roche Stage 2A: diagnosis), and the disease‐progression stage (encompassing Roche Stages 2B through 4B, representing the involvement of additional regions and the need for gastrostomy or respiratory support). Drawing from relevant literature both domestically and internationally and integrating our hospital’s clinical features, specific interventions were developed focusing on the distinct nursing needs across these stages. Tailored nursing measures were formulated based on patient and CITF‐specific considerations encompassing aspects such as self‐concept, disease behavior, and daily life. Main components include (Table 1).

**TABLE 1:** Main contents of nursing intervention based on CITF.

Stage of disease	Correlation dimension	Related nursing interventions
Disease‐onset stage	Self‐concept	Employed verbal and behavioral reassurance techniques to gain cooperation from patients and their families, stabilize patient emotions, and facilitate acceptance of the disease onset
	Disease behaviors	Encouraged patients to improve their physical and mental states and actively cooperate with the medical arrangements of medical staff
	Daily life	We encouraged patients to express their inner thoughts and endeavored to understand their genuine feelings. Subsequently, we analyzed potential reasons for their current psychological state and implemented targeted interventions such as providing emotional support and psychological counseling. We sought to enhance communication with family members, encouraging them to refrain from conveying negative emotions and deliver comprehensive care for the ALS patients

Disease‐diagnosis stage	Self‐concept	We assessed the physical and mental states of ALS patients and guided them to develop accurate perceptions of the disease, aiming to enable them to positively confront changes in physical, psychological, and social aspects. We corrected misconceptions among patients, such as feelings of worthlessness and incapability. We encouraged them to overcome negative emotions and stress through behavioral and motivational training
	Disease behaviors	During this period, patients may need to undergo numerous medical examinations. We encouraged them to cooperate with professionals to complete these examinations. Following a definitive diagnosis, we educated patients about relevant health knowledge related to ALS through video presentations, ALS health manuals, and lectures. This included ALS pathogenesis, diagnostic criteria, treatment, and prognosis. We emphasized the importance of adhering to medical advice to enhance their awareness of the disease
	Daily life	We tried to establish a positive doctor–patient relationship that effectively alleviates their confusion. We made exercise plans based on their personal physical condition and illness trajectory. We ensured the continuity of the CITF management model and regularly adjusted intervention measures

Disease‐progression stage:	Self‐concept	Helped patients adjust to living with the disease and fostered the development of a positive self‐concept. Through constructive communication, assisted patients in understanding the disease appropriately and offered suggestions for effective coping strategies. Patients experiencing intense negative emotions require enhanced psychological interventions, including tailored emotional support
	Disease behaviors	We provided continuous intervention to patients through WeChat. A dedicated WeChat account was applied for follow‐up and guidance. To protect patient privacy, a separate WeChat group was set up for each patient’s rehabilitation care. The group consisted of an attending physician, a rehabilitation physician, a psychologist, and two nurses. We answered patients’ questions online, provided medication guidance, and informed them of follow‐up appointments. In addition, the patients’ vital signs, hemodynamics, and airway patency were comprehensively evaluated weekly, and targeted respiratory function training was conducted with continuous guidance and supervision, including abdominal breathing, pursed‐lip breathing, and diaphragm pacing
	Daily life	We tailored specific interventions for patients, such as listening to music based on personal preferences. We developed a suitable exercise plan for patients. For patients who were self‐reliant, we encouraged them to engage in active exercise. For ALS patients in critical condition, daily exercises in bed for joint mobility, such as joint flexion and extension, hip flexion, and ankle dorsiflexion, were prescribed under the assessment of the rehabilitation physician. Massage both lower limbs daily to prevent muscle spasms and atrophy. Patients were given active swallowing function training, such as cold stimulation, oral exercise, and pronunciation training. In addition, through the WeChat platform, we facilitated communication and encouragement among patients and ensured the continuity of the CITF management model

### 3.1. Observation Indicators

1. Self‐Rating Anxiety Scale (SAS) [16]: This scale comprises 20 items, each rated on a scale of 1–4 points. A total score exceeding 50 is considered indicative of anxiety, with higher scores reflecting more severe anxiety symptoms. Scores derived from the SAS have demonstrated correlation with clinical assessments across various studies, with a Cronbach’s *α* coefficient of 0.879.
2. Self‐Rating Depression Scale (SDS) [17]: The scale consists of 20 items, each evaluated on a range from 1 to 4 points; scores exceeding 53 on the SDS are indicative of depression, with higher scores reflecting more severe depressive symptoms. The SDS is commonly employed to evaluate depressive symptoms and is known for its strong internal consistency and test‐retest reliability, indicating that SDS scores tend to remain stable across various populations. The Cronbach’s *α* coefficient for this scale is 0.806.
3. Mental toughness assessment: The Connor‐Davidson Resilience Scale (CD‐RISC) [18] was utilized to evaluate patients’ mental resilience, encompassing three dimensions with a total of 25 items, each rated from 0 to 4 points. The overall score varies from 0 to 100 and is positively associated with the degree of mental toughness. This scale has a Cronbach’s *α* coefficient of 0.880, signifying excellent internal consistency.
4. Sleep quality assessment: The Pittsburgh Sleep Quality Index (PSQI) [19] includes 19 self‐assessed items and 5 additional items rated by others. Each item is evaluated on a scale of 0–3, resulting in a total score that can range from 0 to 72. Higher scores reflect worse sleep quality. The Cronbach’s *α* coefficient for this scale is 0.78.
5. ALSFRS‐R: The ALS Functional Rating Scale‐Revised (ALSFRS‐R) [20] includes 12 items across four domains of bodily function: bulbar, fine motor, gross motor, and respiratory. Each item is rated on a scale from 0 (*indicating complete loss of function*) to 4 (*indicating no loss of function*), resulting in a total score ranging from 0 to 48. Higher scores signify improved functional ability.
 The patients’ anxiety, depression, mental toughness, and sleep quality were assessed by psychologists. ALSFRS‐R scores were evaluated by 2 neurologists with more than 5 years of clinical experience.

### 3.2. Statistical Analysis

SPSS 25.0 statistical software (IBM, Armonk, NY, USA) was used for data analysis. Categorical data were expressed as frequencies and percentages and analyzed using the chi‐square test. Continuous data were expressed as mean ± standard deviation, and a T‐test was used for comparison between the two groups. *p* < 0.05 was considered statistically significant.

## 4. Results

### 4.1. Patient Characteristics

The study comprised a total of 80 patients, with 40 assigned to the intervention group and 40 to the control group. No statistically significant differences were found between the two groups regarding age, gender, smoking, alcohol, hypertension, diabetes, or coronary heart disease (Table 2) (*p* > 0.05).

**TABLE 2:** Characteristics of the patients in two groups.

Variables	Intervention group (*n* = 40)	Control group (*n* = 40)	*t*/*x* ^2^	*p*
Gender: male	24	18	1.805	0.179
Age, y	53.58 ± 5.34	52.65 ± 6.15	0.718	0.475
Smoking	11	12	0.061	0.805
Alcohol	15	17	0.208	0.648
Hypertension	17	18	0.051	0.822
Diabetes	9	16	2.851	0.091
Coronary heart disease	9	12	0.581	0.446
Riluzole treatment	5	7	0.392	0.531
Edaravone treatment	27	26	0.056	0.813

*Note:* Smoking status: Participants were classified as “current smokers,” “former smokers,” or “never smokers” based on their self‐report during a structured interview. “Current smokers” were defined as those who smoked at least one cigarette per day in the past 30 days. Alcohol use: defined as the consumption of any alcoholic beverage at least once per week on average during the past 30 days. This information was ascertained via a structured questionnaire. Hypertension: defined as either (a) a self‐reported previous diagnosis by a physician; (b) current use of antihypertensive medication; or (c) measured systolic blood pressure ≥ 140 mmHg or diastolic blood pressure ≥ 90 mmHg on at least two occasions at enrollment. Data were sourced from both patient interviews and medical records. Diabetes: defined as (a) a self‐reported physician diagnosis; (b) current use of insulin or oral hypoglycemic agents; or (c) a fasting plasma glucose level ≥ 7.0 mmol/L at enrollment. Data were obtained from medical records and laboratory reports. Coronary heart disease (CHD): defined as a documented history of myocardial infarction, coronary artery bypass grafting, percutaneous coronary intervention, or a diagnosis of angina pectoris confirmed by a cardiologist. This information was exclusively extracted from medical records.

### 4.2. Comparison of Negative Emotional Levels Between Two Groups of Patients

Prior to the intervention, there were no significant differences in the SAS and SDS scores between the two groups (*p* > 0.05). After 8 weeks of nursing intervention, patients in the intervention group showed significantly lower anxiety scores (49.73 ± 1.18) compared to the control group (54.85 ± 3.24) (*p* < 0.05). In addition to anxiety scores, depression scores in the intervention group (48.63 ± 1.17) were lower than those in the control group (52.33 ± 1.12) (Table 3) (*p* < 0.05).

**TABLE 3:** Comparison of negative emotional levels between two groups (points).

Group	*n*	Anxiety scores		Cohen’s *d*_*p*_ (95% CI)	Depression scores		Cohen’s *d*_*p*_ (95% CI)
		Pre‐intervention	Post‐intervention 8 weeks		Pre‐intervention	Post‐intervention 8 weeks	
Intervention group	40	58.50 ± 2.64	49.73 ± 1.18	3.39 (2.58, 4.20)	55.00 ± 1.91	48.63 ± 1.17	2.71 (2.03, 3.38)
Control group	40	59.25 ± 2.92	54.85 ± 3.24	1.54 (1.08, 2.00)	54.90 ± 2.17	52.33 ± 1.12	0.94 (0.56, 1.31)
*T*		1.204	9.407		0.219	14.459	
*p*		0.232	*p* < 0.01		0.827	*p* < 0.01	

### 4.3. Comparison of Mental Toughness Between Two Groups of Patients

Prior to the nursing intervention, there were no significant differences in mental toughness scores (57.45 ± 3.19 vs. 58.15 ± 2.56). After the intervention, the levels of mental toughness increased. Compared to the control group (64.55 ± 1.40), patients in the intervention group exhibited higher mental toughness scores at 8 weeks post‐intervention (72.08 ± 3.93) (Table 4) (*p* < 0.05).

**TABLE 4:** Comparison of mental toughness scores between two groups (points).

Group	*n*	Mental toughness scores		Cohen’s *d*_*p*_ (95% CI)
		Pre‐intervention	Post‐intervention 8 weeks	
Intervention group	40	57.45 ± 3.19	72.08 ± 3.93	−4.14 (−5.10, −3.17)
Control group	40	58.15 ± 2.56	64.55 ± 1.40	−2.60 (−3.25, −1.95)
*T*		1.084	11.426	
*p*		0.282	*p* < 0.01	

### 4.4. Comparison of Sleep Quality Between Two Groups of Patients

Compared to the control group (10.90 ± 1.32), patients in the intervention group exhibited higher sleep quality at 8 weeks postnursing intervention (6.03 ± 0.95) (Table 5) (*p* < 0.05).

**TABLE 5:** Comparison of sleep quality between two groups (points).

Group	*n*	Sleep quality scores		Cohen’s *d*_*p*_ (95% CI)
		Pre‐intervention	Post‐intervention 8 weeks	
Intervention group	40	18.75 ± 1.17	6.03 ± 0.95	8.78 (6.81, 10.74)
Control group	40	18.90 ± 1.32	10.90 ± 1.32	4.16 (3.19, 5.13)
*T*		0.538	19.011	
*p*		0.592	*p* < 0.01	

### 4.5. Comparison of ALSFRS‐R Scores Between Two Groups of Patients

After 8 weeks, ALSFRS‐R scores declined in both groups, with the control group exhibiting significantly lower scores compared to the intervention group (Table 6) (*p* < 0.05).

**TABLE 6:** Comparison of ALSFRS‐R scores between two groups of patients (points).

Group	*n*	ALSFRS‐R scores		Cohen’s *d*_*p*_ (95% CI)
		Pre‐intervention	Post‐intervention 8 weeks	
Intervention group	40	33.43 ± 3.04	31.63 ± 3.54	1.38 (0.94, 1.81)
Control group	40	33.65 ± 3.91	29.58 ± 3.38	2.73 (2.05, 3.41)
*T*		0.288	2.648	
*p*		0.774	0.010	

## 5. Discussion

Our study demonstrated that constructing nursing interventions based on CITF significantly alleviated anxiety and depression symptoms in ALS patients. The CITF management mode enhanced patient mental toughness and sleep quality, contributing to increased quality of life. Our research suggests that the CITF management mode may be a new direction for nursing care in ALS.

Chronic disease is known to gradually worsen over the years. While the ultimate outcome of chronic diseases cannot be altered, effective nursing interventions should be implemented to slow the progression of the disease and maintain good health and quality of life. Traditional nursing often provides written or verbal instructions when patients seek medical care. However, this results in reduced treatment adherence among patients [21]. After the onset, individuals with ALS may experience severe disabilities within months or years, often necessitating assistance in their daily lives. However, there is currently a lack of effective ALS treatments [22, 23]. Consequently, as the disease progresses, the associated decline in functional status and changes in social roles often lead patients to experience severe anxiety and depressive states [24]. Additionally, many patients, due to the rapid progression of ALS, develop severe speech impairments, making it difficult to express daily needs. Consequently, their inner thoughts and feelings become incomprehensible to those around them, exacerbating negative emotions. Related studies indicate that the comorbid prevalence of depression in patients with ALS is 34%, with prevalence rates of 16% for moderate and 8% for severe depression [25]. The concept of CITF was initially proposed by Strauss, suggesting that chronic illnesses exhibit multidimensional and variable disease processes. By tailoring interventions based on disease progression characteristics, personalized approaches can assist chronic patients in managing disease progression and improving quality of life. Our study indicated that after nursing intervention, SDS and SAS scores in ALS patients gradually decreased, with a more pronounced decline observed in the CITF intervention group. Previous research has shown [26] that applying CITF intervention in the nursing care of glioma patients postradiotherapy significantly enhances patients’ psychological resilience, regulates negative emotions, and reduces negative behaviors. Consistent with this study, our research found that the CITF management mode also improves negative emotions in ALS patients.

Mental toughness, as a psychological construct, was initially applied in the field of sports. It refers to a stable personality trait that enables individuals to consistently perform at their best, even under high‐pressure situations [27]. Wagnild et al. [28] developed the Resilience Scale (RS), which categorizes resilience into three dimensions: competence, self, and acceptance of life. Building upon this framework, CD‐RISC is based on capabilities and traits, which has become the most widely used clinical assessment tool. Higher scores indicate greater mental toughness in patients, correlating with fewer psychological health issues. Robust mental toughness enables patients to face illnesses and surgeries with a more positive mindset [26]. Our study demonstrated that after intervention, ALS patients showed increased scores in CD‐RISC, with a more significant increase observed in the CITF intervention group. This indicated that the CITF management mode significantly enhanced the level of mental toughness in ALS patients. Additionally, we found that the CITF management mode effectively improved patients’ sleep quality.

The management mode based on CITF emphasizes personalized needs. We offered continuous nursing support through WeChat to ensure individualized care. Our intervention protocol in this study provided precise disease knowledge, psychological interventions, and behavioral interventions tailored to patients from the onset to the progressive stages of ALS. Each stage focused on specific aspects. The interventions were specific, rather than applying uniform approaches to address existing issues. We formulated individualized rehabilitation measures according to the specific condition of each patient, which significantly increased the ALSFRS‐R scores and improved the quality of life. The significant improvement observed in our study can be attributed to this structured, CITF‐based approach. By delivering stage‐specific interventions, such as providing psychological support in the early stages and implementing comprehensive remote monitoring along with personalized respiratory and swallowing training in the progressive stage, we optimized patients’ functional status and mitigated complications, ultimately fostering greater patient independence and active participation in their care.

Several limitations need to be considered in this study. Firstly, the sample size was limited and derived from hospitals in a single region, which could restrict the broader applicability of the study’s findings. Furthermore, no subgroup analysis was conducted on patients at different stages. Large‐scale, multicenter studies should be conducted in the future. Although the CITF management mode was employed, detailed procedures were not provided, which could impact the replicability of the intervention in other hospitals. Additionally, the study duration was relatively short, and continuous nursing care for ALS patients until the terminal stage was not provided, thus lacking long‐term intervention effects. Ultimately, our assessment instruments, including the SDS, SAS, and CD‐RISC, were based on patient self‐reports, which introduces the potential for respondent bias and subjectivity. We acknowledge these limitations and encourage future studies to adopt broader and more diverse populations, as well as employ measurement methods aimed at mitigating biases.

Above all, the CITF management mode can ameliorate anxiety and depression in ALS patients and enhance their mental toughness and sleep quality. This personalized and comprehensive care strategy not only improves treatment compliance but also enhances overall quality of life. By focusing on individual needs and adopting a holistic approach to patient care, healthcare interventions can be enhanced to promote more effective health management. The uniqueness of this study lies in its pioneering application of the CITF management model in ALS patients in China. This innovative approach is expected to positively impact the healthcare sector in China, offering a novel pathway for improving health management in this population. Future research could investigate the relevance of the CITF model across various patient populations, considering factors such as age, gender, and cultural background.

## 6. Relevance for Clinical Practice

Implementing CITF‐based nursing interventions enables nurses to effectively address the complex and evolving needs of ALS patients, ensuring culturally competent and personalized care. Nurses should strive to integrate the CITF into their daily practice and use the insights gained to inform their care strategies. To successfully adopt CITF‐based care, education and theoretical training should be complemented with hands‐on clinical experience and continuous feedback. Nurses should focus on understanding the distinct emotional and functional challenges faced by ALS patients at each stage of the disease. Developing a habit of seeking feedback from patients, their families, and interdisciplinary team members can help nurses refine their interventions and enhance their responsiveness to patient needs. Additionally, nurses can engage in reflective practice at the end of each shift, documenting the challenges encountered, their responses, and the effectiveness of the strategies employed. This reflection enables nurses to incorporate their learnings into future practice, thereby improving the quality of care provided. By adopting CITF‐based interventions, nurses can reduce patients’ anxiety and depression, enhance mental resilience, improve sleep quality, and maintain better functional status, ultimately leading to an improved quality of life for ALS patients.

## 7. Conclusion

In conclusion, this prospective study demonstrates the significant clinical utility of nursing interventions based on the CITF in the holistic management of ALS. By implementing staged, individualized, and multidimensional care, this framework addresses multifaceted needs—including illness‐related, biographical, and everyday living aspects. This approach effectively alleviated patient anxiety and depression, enhanced psychological resilience and sleep quality, and was associated with improved functional status as indicated by ALSFRS‐R scores. These findings underscore the utility and pertinence of the CITF in addressing the complex, evolving needs of patients with ALS over time. While future multicenter, longitudinal studies are warranted to further validate this model and optimize intervention protocols, integrating the CITF into standard ALS care practices represents a promising strategy for improving emotional well‐being, functional status, and overall quality of life.

## Author Contributions

Jiao Zhen: conceptualization, writing–original draft, and writing–review and editing; Qia Liu: writing–review and editing; Xiaolu Xue: methodology and data curation; Fengjiao Hao: methodology and software; Shufang Zhang: software and data curation; Na Liu: formal analysis and writing–original draft; Zixuan Li: investigation and data collection; Jing Chen: validation; Junxiang Cheng: conceptualization, resources, supervision, project administration, and funding acquisition.

## Funding

This study was not supported by any funding.

## Ethics Statement

Ethical approval for this study was obtained from the Ethics Committee of the First Hospital of Shanxi Medical University (Approval No. KYLL‐2024‐258).

## Consent

Informed consent was obtained from all individual participants included in the study.

## Conflicts of Interest

The authors declare no conflicts of interest.

## Data Availability

The data that support the findings of this study are available from the corresponding author upon reasonable request.
